# ZEB1-mediated suppression of circDennd1b promotes the migration and invasion of lung cancer cells

**DOI:** 10.1080/19768354.2026.2742602

**Published:** 2026-10-06

**Authors:** Seongran Cho, Jin-A Choi, Jihye Park, Keunsoo Kang, Young-Ho Ahn

**Affiliations:** a Department of Molecular Medicine, Inflammation-Cancer Microenvironment Research Center, College of Medicine, Ewha Womans University, Seoul, Korea; b Department of Biomedical Sciences, College of Bio-Convergence, Dankook University, Cheonan, Korea

**Keywords:** ZEB1, circular RNA, circDennd1b, epithelial–mesenchymal transition, miR-181

## Abstract

Zinc finger E-box binding homeobox 1 (ZEB1) is a key transcription factor that induces the epithelial–mesenchymal transition (EMT) and promotes lung cancer progression. While circular RNAs (circRNAs) are also involved in EMT, their connection with ZEB1 remains unclear. Here, using RNA sequencing coupled with CIRI-full analysis, we identified 25 circRNAs differentially expressed in ZEB1-overexpressing lung cancer cells. Among them, *circDennd1b* was significantly downregulated. *circDennd1b* knockdown promoted cell proliferation, migration, and invasion, and induced EMT. Conversely, *circDennd1b* overexpression inhibited cancer cell migration and invasion. We also found that *circDennd1b* inhibits cell migration by sponging the miR-181 family. These data indicate that the ZEB1/*circDennd1b*/miR-181 axis promotes lung cancer progression and could offer a new approach for treatment.

## GRAPHICAL ABSTRACT

**Figure:**
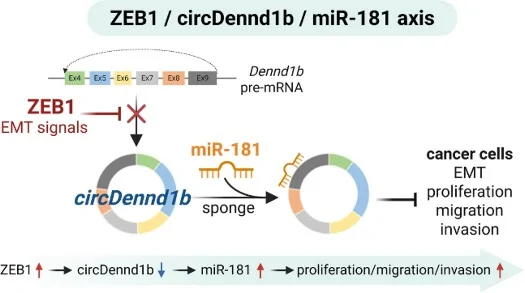

## Introduction

Circular RNAs (circRNAs) have emerged as critical regulators of cancer progression, particularly through their interactions with epithelial–mesenchymal transition (EMT) signaling pathways (Shang et al. 2019). EMT is a dynamic process where epithelial cells acquire mesenchymal traits, enhancing their invasive and metastatic potential (Kalluri and Weinberg 2009). Key signaling pathways, including TGFβ, Wnt/β-catenin, and Notch, drive this phenotypic shift while also influencing circRNA expression and activity, suggesting a complex bidirectional relationship. TGFβ, a potent EMT inducer, promotes the expression of *circRNA_0006508* in hepatocellular carcinoma. This circRNA functions as a molecular sponge for miR-125a/miR-125b, effectively reducing their inhibition of EMT-related transcription factors such as SNAIL1. Consequently, this interaction enhances cancer cell migration and invasion (Deshmukh et al. 2021; Papatsirou et al. 2021). Similarly, Wnt/β-catenin signaling enhances *circFoxo3* expression, which regulates the cell cycle and apoptosis to support EMT and increase cancer invasiveness (Xu et al. 2020; Papatsirou et al. 2021). In breast cancer, Notch signaling upregulates *circRNA_0001785*, which sponges miR-326 to promote EMT by modulating Notch pathway genes, driving cancer cell proliferation and migration (Deshmukh et al. 2021; Papatsirou et al. 2021). These examples underscore the interplay between EMT pathways and circRNA networks in shaping aggressive cancer phenotypes.

ZEB1, a key regulator of EMT, controls circRNA biogenesis through transcriptional and post-transcriptional mechanisms. In bladder cancer, ZEB1 promotes *circNIPBL* formation by binding to intronic regions of the *NIPBL* gene, establishing a positive feedback loop via the *circNIPBL*/miR-16-3p/Wnt5a/ZEB1 axis to sustain metastasis (Li et al. 2023). In breast cancer, ZEB1 binds to the promoter of *WWC3*, which increases the expression of *circWWC3*. This circular RNA serves as a molecular sponge for miR-26b-3p and miR-660-3p to activate the Ras signaling pathway, such as EGFR and MAPK1 (Meng et al. 2020). In contrast, ZEB1 recruits the RNA-binding protein EIF4A3 to reduce *circDOCK5* formation in esophageal squamous cell carcinoma. This disrupts the miR-627-3p/TGF-β signaling and promotes metastasis (Meng et al. 2021). Also, the ZEB1/miR-200 axis controls the biogenesis of EMT-associated circRNAs by regulating the RNA-binding protein QKI (Conn et al. 2015; Kim et al. 2019). These studies show that ZEB1 is a key regulator of circRNAs during cancer progression.

Despite these findings, the role of ZEB1-regulated circRNAs in lung cancer is still unclear. In this study, we identified *circDennd1b* as a circular RNA suppressed by ZEB1 in lung cancer cells. Previous studies showed *circDennd1b* promotes cholesterol efflux by sponging miR-17-5p in atherosclerosis (Xu et al. 2021) and inhibits tumorigenicity by sponging miR-122-5p in clear cell renal cell carcinoma (Chen et al. 2022). However, its connection to EMT in lung cancer has not been investigated. Here, we show that *circDennd1b* suppresses lung cancer cell migration and invasion by sponging miR-181 family. This ZEB1/*circDennd1b* axis contributes to lung cancer progression and could be considered for future therapies.

## Materials and methods

### Cell culture

393P, 344SQ, and ZEB1-overexpressing 393P (393P-ZEB1) cell lines were cultured in RPMI 1640 (Welgene; Gyeongsan, Korea) supplemented with 10% FBS (Corning; Corning, NY, USA) at 37°C with 5% CO_2_. To induce EMT, 393P cells were treated with 5 ng/mL TGFβ for 7 days. For cell growth assays, cells were seeded in 96-well plates (2 × 10^3^ cells/well), and cell viability was measured daily for 3-4 days using the WST-8 reagent (Biomax; Guri, Korea).

### RNA sequencing

Total RNA was isolated in triplicate from 393P-vec and 393P-ZEB1 cell lines utilizing the XENOPURE Total RNA Purification Kit (Xenohelix; Incheon, Korea). Following extraction, samples were transferred to Macrogen (Seoul, Korea) for transcriptome analysis. RNA quality and concentration were verified before library preparation using the TruSeq Stranded Total RNA Library Prep Gold Kit (Illumina; San Diego, CA, USA). Sequencing was executed on an Illumina NovaSeq platform, generating paired-end reads. For circRNA identification, circRNA-seq reads were mapped to the GRCm38/mm10 mouse genome with BWA-MEM v0.7.17 (Li 2013). We used CIRI-full v2.0 (Zheng et al. 2019) to detect circRNAs, as it integrates CIRI2 for back-splicing junction (BSJ) detection with CIRI-AS for isoform reconstruction. A minimum of 10 BSJ-spanning reads was required per circRNA candidate. BSJ structures were checked with CIRI-vis. Expression of circRNAs was quantified by CIRIquant (Zhang, Chen, et al. 2020). Genomic annotations were added using HOMER v4.11 (Heinz et al. 2010), and differential expression between groups was tested from BSJ count matrices using Python 2.7. The RNA-seq raw data and processed files have been deposited in NCBI’s Gene Expression Omnibus (GSE319378).

### Knockdown and overexpression of *circDennd1b* and miR-181c/d

To knockdown *circDennd1b*, a custom siRNA targeting the back-splice junction (5′-GAGCAAAUUAAGCACUGAA-3′) was synthesized by Bioneer (Daejeon, Korea) and transiently transfected into 393P cells using the TransIT-X2 Dynamic Delivery System (Mirus Bio; Madison, WI, USA). For stable knockdown, an shRNA oligomer featuring the same target sequence was cloned into the pLKO.1-Puro lentiviral vector (Addgene #10878). miR-181c/d inhibitors were also obtained from Bioneer and transiently transfected using the TransIT-X2 system. To generate the *circDennd1b* expression construct, exons 4–9 of murine *Dennd1b* were first PCR-cloned into the pcDNA3.1(+) ZKSCAN1-MCS Exon Vector (Addgene #69901). The resulting cassette, including the flanking intronic repeats required for efficient circularization, was subcloned into the pLVX-Puro lentiviral vector (Clontech; Mountain View, CA, USA). To overexpress miR-181c/d, a genomic region (846 bp) containing the pre-miR-181c/d cluster was PCR-amplified from 344SQ murine lung cancer cells and cloned into the pLVX-Puro vector. Lentiviral particles were then produced in 293T cells via co-transfection of the transfer vectors with packaging plasmids pMD2.G (Addgene #12259) and psPAX2 (Addgene #12260) using Lipofector-EXT (AptaBio; Yongin, Korea) (Kim et al. 2025). Target cells were infected with the viral supernatant and stably selected using puromycin for at least two weeks before downstream assays.

### Reverse transcription-PCR (RT–PCR)

Total RNA was isolated using the WelPrep Total RNA Isolation Reagent (Welgene), following the manufacturer’s protocol. For cDNA synthesis, reverse transcription was performed using either TOPscript RT DryMIX (Enzynomics; Daejeon, Korea) or SuperScript IV VILO Master Mix (Invitrogen; Waltham, MA, USA). To check circRNA expression, PCR was performed using Lamp Taq DNA Polymerase (Biofact; Daejeon, Korea) with divergent primers targeting the back-splice junction (Supplementary Table 1). PCR products were run on agarose gels and band densities were quantified using ImageJ. Quantitative RT–PCR (qRT-PCR) was performed using Real-Time PCR Master Mix with SFCgreen I (Biofact), and mRNA levels were normalized to *Rpl32*. All primer sequences are listed in Supplementary Table 1. Also, TaqMan probes (Applied Biosystems; Waltham, MA, USA) were used to measure *circDennd1b* levels. To quantify miRNAs, the HB miR Multi Assay Kit (Heimbiotek; Seongnam, Korea) was used, and data were normalized to *RNU6B* snoRNA.

### Transwell migration assay

Cells (1 × 10^5^ per insert) in serum-free medium were seeded into the upper chambers, and complete medium with 10% FBS was added to the lower chambers of Transwell inserts (Labselect; Hefei, China). After 24 h, the migrated cells stained with crystal violet were photographed and manually counted using ImageJ.

### Three-dimensional (3-D) spheroid invasion assay

Multicellular cancer cell spheroids were generated using agarose micromolds prepared with the 3D Petri Dish micro-mold kit (MicroTissues; Providence, RI, USA). Cells (2.56 × 10^5^ cells per mold) were seeded into 2% agarose micromolds and allowed spheroids to form for 2 days. The harvested spheroids were then embedded in collagen domes (comprising spheroids in 0.5× PBS, 0.01 N NaOH, and 3 mg/mL collagen) at the center of each well in a 12-well plate. After 1-2 days, invasion ratios were quantified as the total invaded area divided by the initial central spheroid area using ImageJ.

### AGO2-RNA immunoprecipitation

RNA immunoprecipitation (RIP) was performed using the EpiNext CUT&LUNCH RIP Kit (EpigenTek; New York, NY, USA) according to the manufacturer’s instructions. Control and miR-181c/d-overexpressing 393P cells were permeabilized and incubated with an AGO2 antibody (EpigenTek). After enzymatic RNA cleavage, AGO2-bound RNA complexes were captured with affinity beads. The RNA was then purified and analyzed for *circDennd1b* levels by qRT-PCR.

### Luciferase assay

Exons 4 through 9 of *circDennd1b* were inserted into the psiCHECK-2 vector (Promega; Madison, WI, USA), and the miR-181-binding site was mutated using PCR-based site-directed mutagenesis. Control or miR-181c/d-overexpressing 393P cells (1 × 10^5^ cells/well) were seeded in 24-well plates. On the next day, the cells were transfected with the reporter constructs using Lipofector-EXT. After 48 h, luciferase activity was measured with the Luc-Pair Duo-Luciferase Assay Kit (GeneCopoeia; Rockville, MD, USA) on an INNO-M microplate luminometer (LTek; Seongnam, Korea).

### Statistics

Statistical analyses were performed using GraphPad Prism (La Jolla, CA, USA). Significant differences between groups were determined using Student’s t-test or two-way ANOVA. Experiments were repeated 3-4 times unless noted otherwise. Statistical significance was set at *P* < 0.05, indicated as *P < 0.05, **P < 0.01, and ***P < 0.001.

## Results

### ZEB1 overexpression downregulates *circDennd1b* in lung cancer cells

ZEB1 overexpression in murine epithelial-like 393P lung cancer cells (393P-ZEB1) induced a mesenchymal-like spindle shape (Figure 1A). ZEB1 overexpression upregulated mesenchymal markers (*Zeb1*, *Twist1*, and *Cdh2*) and downregulated epithelial markers (*Cdh1*, *Crb3*, and *Cldn3*), as previously reported (Kim et al. 2022; Lee et al. 2025). To identify circRNAs regulated by ZEB1, we performed RNA sequencing on 393P-ZEB1 and control cells (393P-vec). CIRI-full analysis (Zheng et al. 2019) revealed 25 circRNAs significantly regulated by ZEB1 (11 upregulated and 14 downregulated; fold-change ≥ 2, *P* ≤ 0.05) (Figure 1B; Supplementary Figure 1A). We then validated the expression of eight candidates by RT–PCR using divergent primers targeting the predicted back-splicing junctions (Figure 1C and D; Supplementary Figure 1B). Among them, *circDennd1b*, which is formed by exons 4 through 9 of *Dennd1b* mRNA, was the most significantly downregulated (Figure 1D and E). Sequencing of the RT–PCR product confirmed the back-splicing junction between Exon 4 and Exon 9 (Figure 1F). The linear *Dennd1b* mRNA level remained unchanged (Figure 1G), suggesting that ZEB1 indirectly regulates *circDennd1b* back-splicing rather than host gene transcription. *circDennd1b* expression was also lower in murine mesenchymal-like 344SQ cells compared with epithelial-like 393P cells (Figure 1H). TGFβ-induced EMT (Kim et al. 2022) similarly reduced *circDennd1b* levels (Figure 1I), suggesting that its downregulation is related to the EMT process.

**Figure 1.**
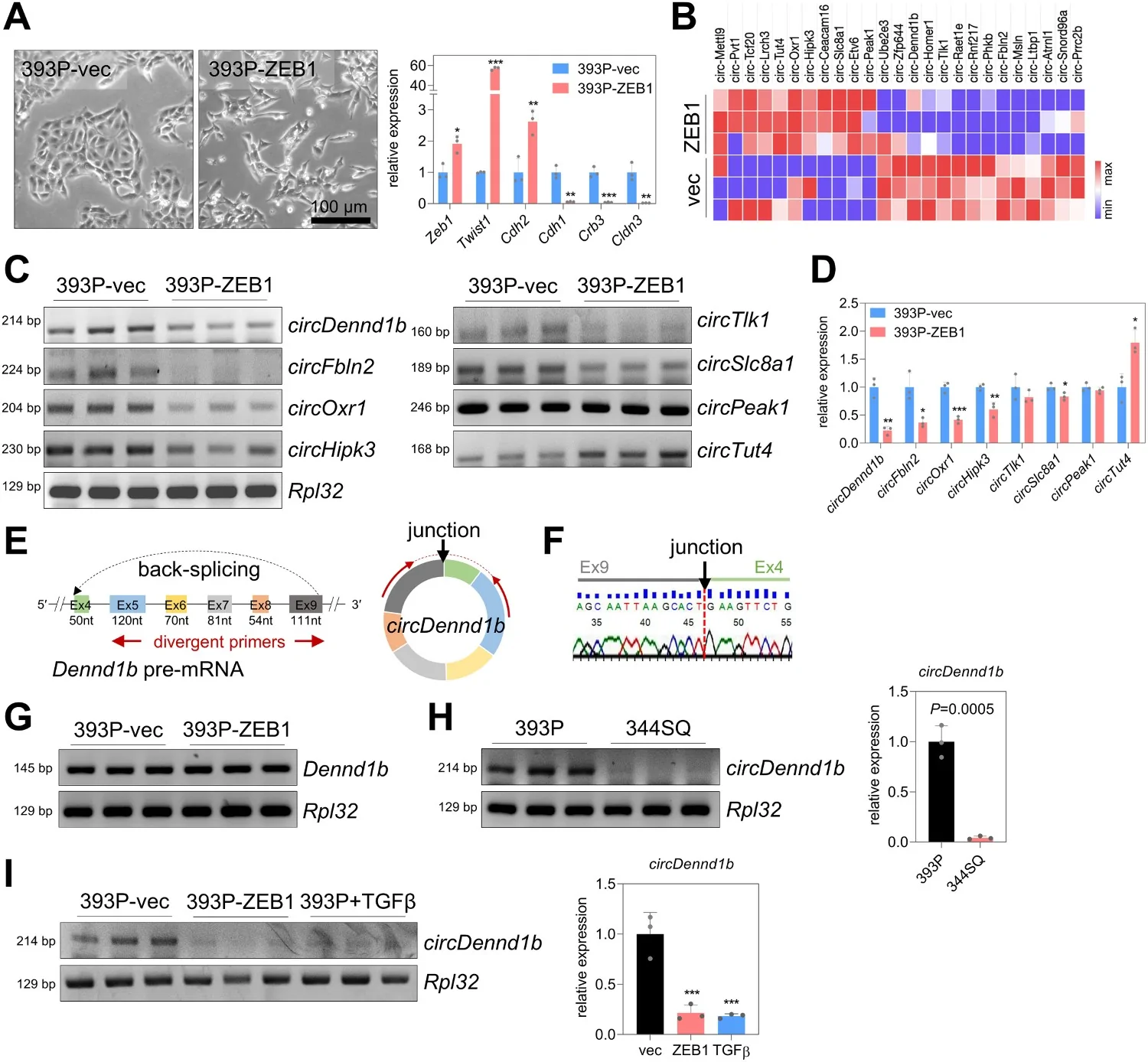
*circDennd1b* is downregulated by ZEB1 and TGFβ during EMT in lung cancer cells. (**A**) Characterization of ZEB1-induced EMT in 393P murine lung cancer cells. Left: Phase-contrast microscopy of 393P cells transfected with control (393P-vec) or ZEB1-overexpression (393P-ZEB1) vectors. Right: qRT-PCR analysis of EMT markers in 393P-vec and 393P-ZEB1 cells; expression was normalized to *Rpl32* mRNA. *P < 0.05, **P < 0.01, and ***P < 0.001 by two-tailed Student’s t-test. (**B**) Heatmap of differentially expressed circRNAs between 393P-vec and 393P-ZEB1 cells. CIRI-full analysis of RNA-seq data identified 25 candidate circRNAs significantly regulated by ZEB1 (fold-change > 2, *P* < 0.05). Red and blue indicate increased and decreased expression, respectively. (**C**) RT-PCR validation of candidate circRNAs in 393P-vec and 393P-ZEB1 cells. *Rpl32* mRNA served as an internal control. (**D**) Quantification of the RT-PCR data shown in Panel C. *P < 0.05, **P < 0.01, and ***P < 0.001 by two-tailed Student’s t-test. (**E**) Schematic diagram illustrating the back-splicing mechanism of *Dennd1b* exons to generate *circDennd1b*. (**F**) Sanger sequencing chromatogram of the *circDennd1b* back-splice junction, confirming the fusion of Exon 9 to Exon 4. (**G**) RT-PCR analysis of linear *Dennd1b* mRNA levels in 393P-vec and 393P-ZEB1 cells. (**H**) RT-PCR analysis of *circDennd1b* expression in epithelial-like 393P and mesenchymal-like 344SQ cells. *P*-value was determined by two-tailed Student’s t-test. (**I**) RT-PCR analysis of *circDennd1b* in 393P cells treated with 5 ng/mL TGFβ for 7 days to induce EMT. ***P < 0.001 by two-tailed Student’s t-test.

### *circDennd1b* knockdown promotes the migration and invasion of lung cancer cells

To study its functional role, we stably knocked down *circDennd1b* in 393P cells using a junction-specific shRNA (Figure 2A), which did not affect *Dennd1b* mRNA levels (Figure 2B). *circDennd1b* knockdown slightly but significantly increased cell growth at day 4 (Figure 2C). It also increased cell migration at 24 h (Figure 2D) and 3-D spheroid invasion at 48 h (Figure 2E). qRT-PCR of EMT markers confirmed that *circDennd1b* knockdown induced EMT (Figure 2F). siRNA-mediated transient knockdown showed similar effects on cellular migration and invasion (Figure 2G–I). Together, these results indicate that *circDennd1b* knockdown enhances lung cancer cell migration and invasion, although this effect might partly result from increased growth.

**Figure 2.**
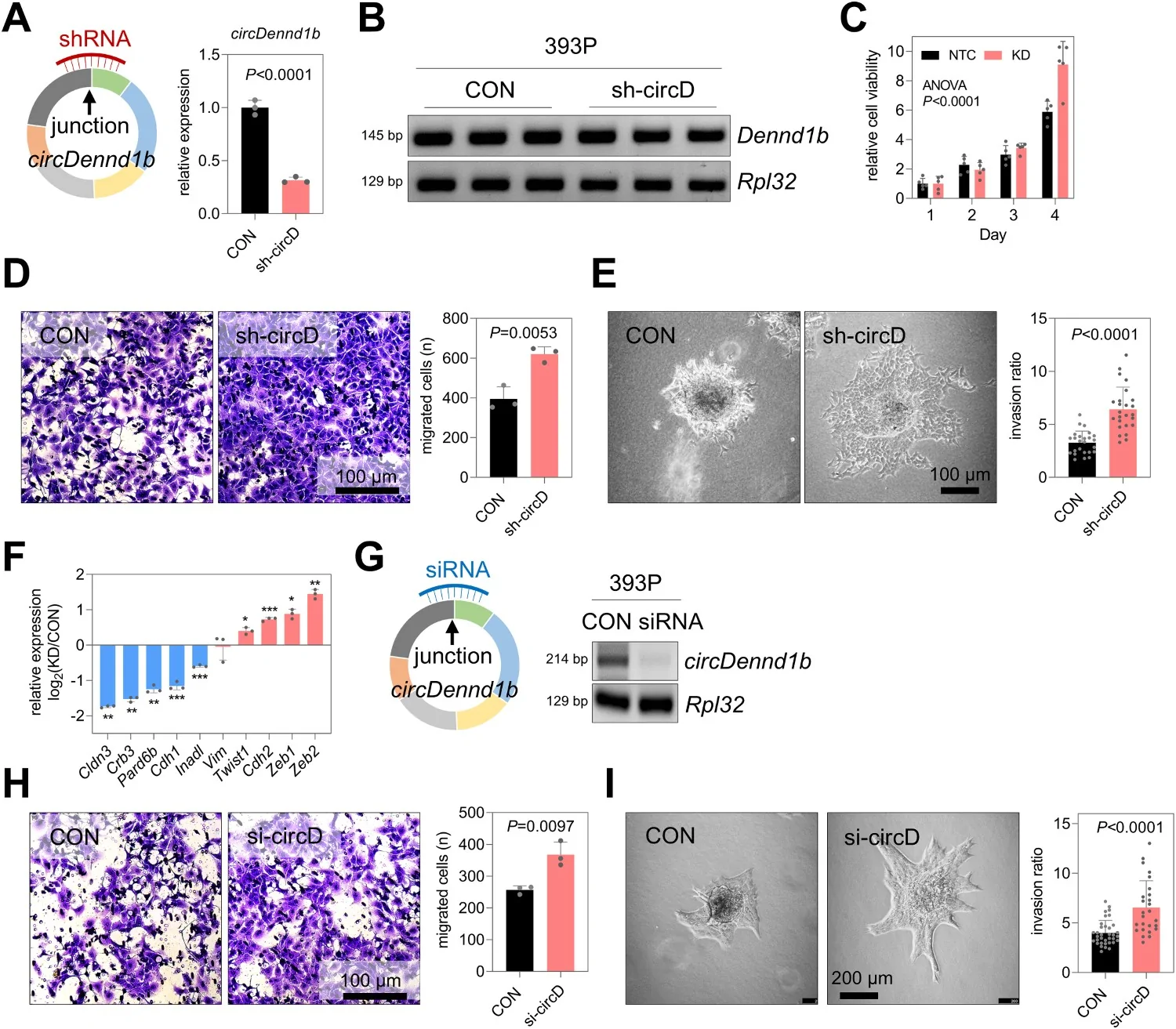
*circDennd1b* knockdown (KD) promotes the migration and invasion of lung cancer cells. (**A**) qRT-PCR analysis of *circDennd1b* expression in 393P cells transduced with control (CON) or *circDennd1b*-targeting shRNA (KD). *P*-value was determined by two-tailed Student’s t-test. (**B**) RT-PCR analysis of linear *Dennd1b* mRNA levels in *circDennd1b* KD cells to confirm knockdown specificity. (**C**) Cell viability assay of 393P-CON and *circDennd1b* KD cells. *P*-value was determined by two-way ANOVA. (**D**) Transwell migration assay of 393P-CON and *circDennd1b* KD cells. Cells were seeded in the upper chambers; after 24 h, migrated cells were fixed, stained with crystal violet, and quantified. *P*-value was determined by two-tailed Student’s t-test. (**E**) Spheroid invasion assay in 393P-CON and *circDennd1b* KD cells. Spheroids were generated using microwells, embedded in collagen gels, and cultured for 48 h. The invasion ratio (total area/central spheroid area) was quantified using ImageJ. *P*-value was determined by two-tailed Student’s t-test. (**F**) qRT-PCR analysis of EMT markers in 393P-CON and *circDennd1b* KD cells. Expression was normalized to *Rpl32* mRNA. Data are presented as log2 fold-change (KD vs. CON). *P < 0.05, **P < 0.01, and ***P < 0.001 by two-tailed Student’s t-test. (**G**) RT-PCR validation of *circDennd1b* levels in 393P cells transfected with control (CON) or *circDennd1b*-specific siRNA. (**H**) Transwell migration assay of 393P cells following siRNA-mediated knockdown of *circDennd1b*. *P*-value was determined by two-tailed Student’s t-test. (**I**) Spheroid invasion assay of 393P cells following siRNA-mediated knockdown of *circDennd1b*. *P*-value was determined by two-tailed Student’s t-test.

### *circDennd1b* overexpression suppresses the migration of lung cancer cells

We next overexpressed *circDennd1b* in 393P-ZEB1 cells using a lentiviral vector modified from the ZKSCAN1-MCS system, which contains intronic repeats to ensure efficient circRNA production (Figure 3A) (Kramer et al. 2015). This viral transduction successfully increased *circDennd1b* levels without affecting the expression of its linear mRNA counterpart, *Dennd1b* (Figure 3B). Overexpression of *circDennd1b* significantly suppressed the growth and migration of 393P-ZEB1 cells (Figure 3C and D). Furthermore, *circDennd1b* overexpression suppressed the expression of mesenchymal markers (Figure 3E). These results indicate that *circDennd1b* suppresses EMT and lung cancer cell migration.

**Figure 3.**
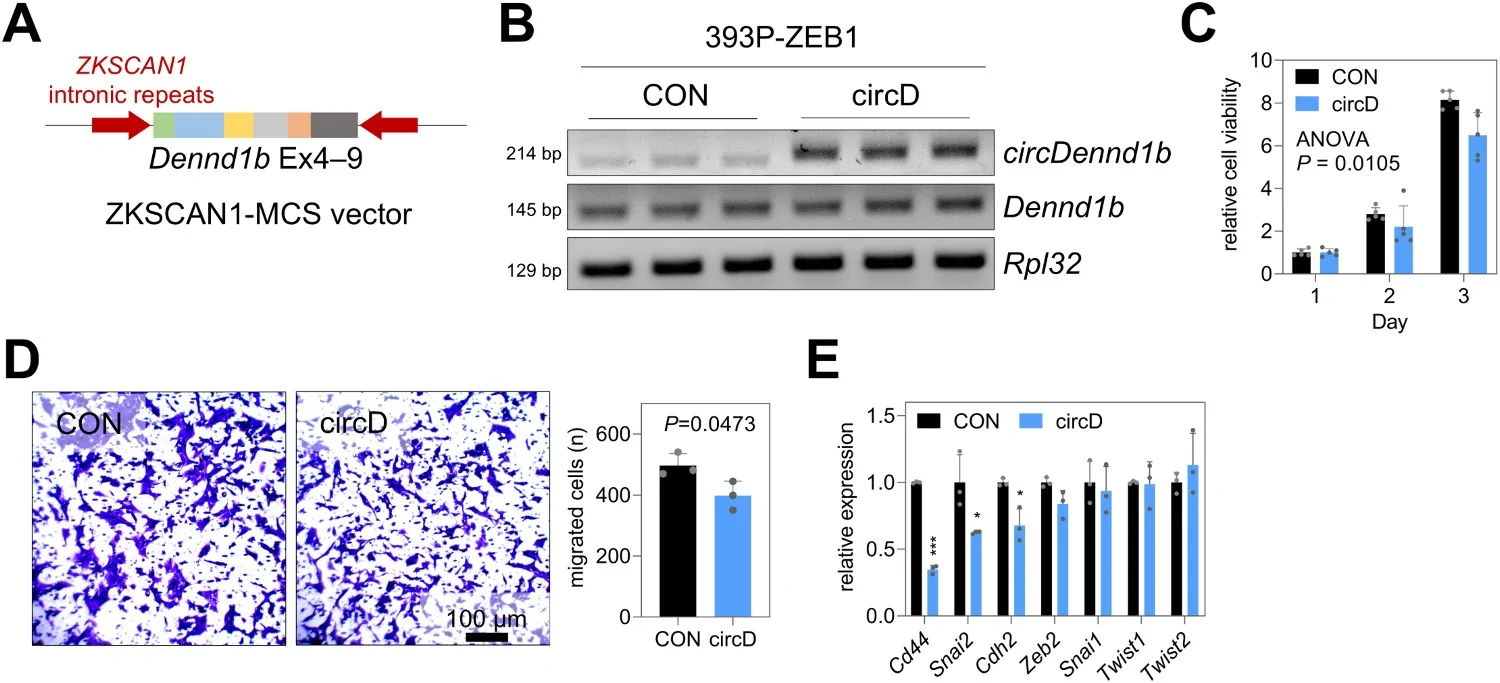
*circDennd1b* overexpression inhibits the migration of lung cancer cells. (**A**) Schematic diagram illustrating the construction of the *circDennd1b* overexpression vector. (**B**) RT-PCR analysis of *circDennd1b* and linear *Dennd1b* mRNA levels in 393P-ZEB1 cells transduced with either a control (CON) or *circDennd1b* overexpression (OE) vector. *Rpl32* mRNA served as an internal control. (**C**) Cell viability assay of 393P-ZEB1-CON and *circDennd1b* OE cells. Statistical significance was determined by two-way ANOVA. (**D**) Transwell migration assay comparing 393P-ZEB1-CON and *circDennd1b* OE cells. *P*-value was calculated using a two-tailed Student’s t-test. (**E**) qRT-PCR analysis of mesenchymal markers in 393P-ZEB1-CON and *circDennd1b* OE cells.

### *circDennd1b* functions as a sponge of miR-181 family

CircRNAs often act as miRNA sponges to regulate gene expression (Choi et al. 2022; Hwang et al. 2025; Yoon et al. 2025). To find miRNAs sponged by *circDennd1b*, we used the circBank database (Liu et al. 2019) and our previous miRNA profiling data (NCBI-GEO GSE38386) (Ahn et al. 2012). Among the predicted targets, the miR-181 family (miR-181a, miR-181c, and miR-181d) was significantly upregulated in 393P-ZEB1 cells (Figure 4A). miR-181 levels increased after *circDennd1b* knockdown and decreased after its overexpression (Figure 4B and C). This suggests that *circDennd1b* may act as a partial sponge for the miR-181 family in our experimental conditions. Ectopic miR-181c/d expression increased 393P cell growth (Figure 4D and E) and motility (Figure 4F). In contrast, miR-181c/d inhibition reduced migration in 393P-ZEB1 or *circDennd1b* knockdown cells (Figure 4G and H; Supplementary Figure 2). Finally, AGO2-RIP and luciferase reporter assays confirmed that *circDennd1b* interacts with miR-181 (Figure 4I and J). Overall, *circDennd1b* may attenuate cell migration, partly by sponging the pro-migratory miR-181 family.

**Figure 4.**
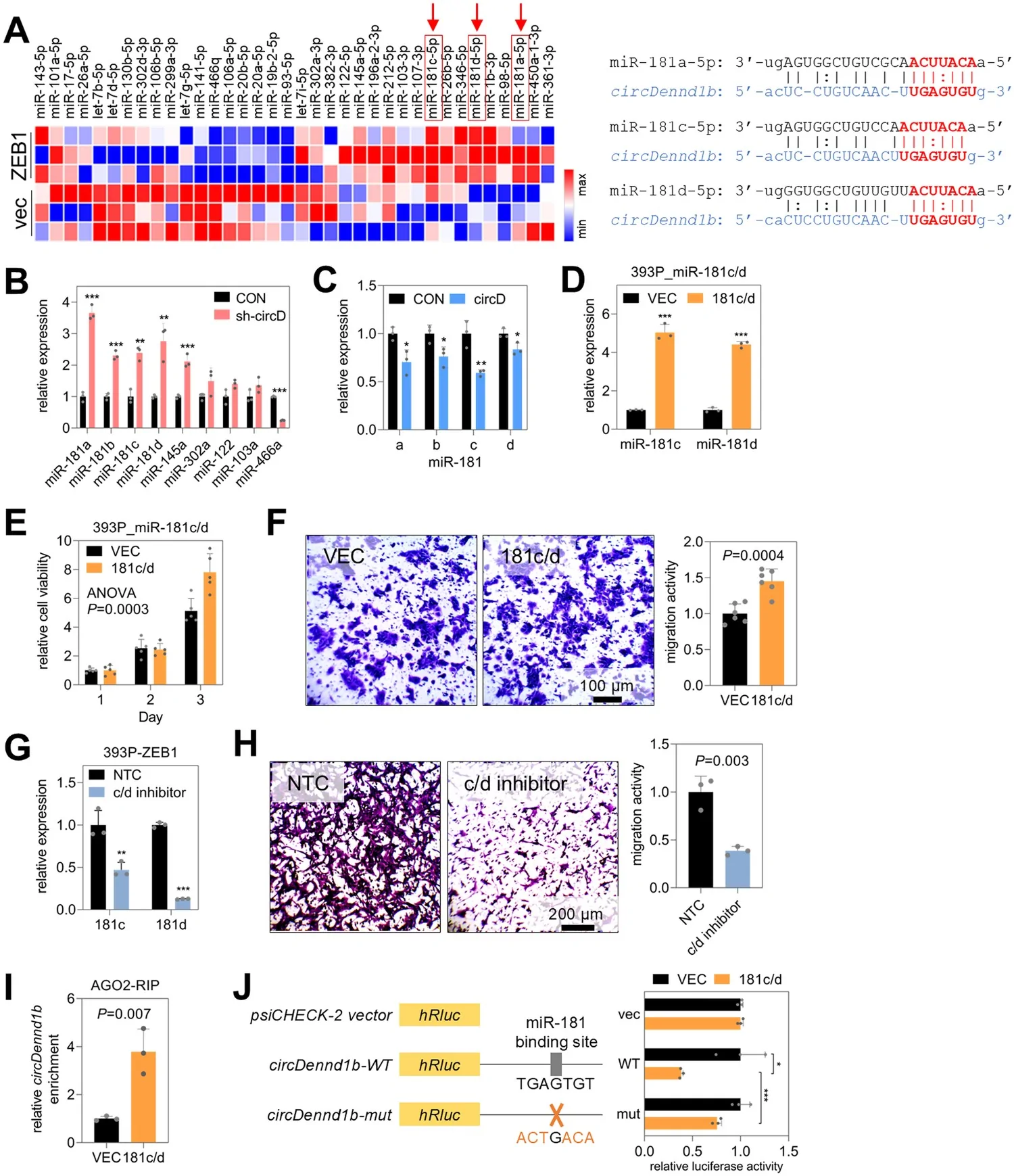
*circDennd1b* acts as a sponge for the miR-181 family. (**A**) Left: Heatmap of miRNA profiling in 393P-vec and 393P-ZEB1 cells, highlighting candidate miRNAs predicted to be sponged by *circDennd1b* via the circBank database. Right: Schematic representation of the predicted binding sites between *circDennd1b* and miR-181 family members. (**B**) qRT-PCR analysis of miRNA expression levels in 393P-CON and *circDennd1b* knockdown (KD) cells. miRNA levels were normalized to *RNU6B* snoRNA; data are presented relative to 393P-CON (set to 1.0). **P < 0.01 and ***P < 0.001 by two-tailed Student’s t-test. (**C**) qRT-PCR analysis of miR-181 family expression in 393P-ZEB1-CON and *circDennd1b* overexpressing (OE) cells. *P < 0.05 and **P < 0.01 by two-tailed Student’s t-test. (**D**) qRT-PCR validation of miR-181 family levels in 393P-vec and miR-181c/d OE cells. ***P < 0.001 by two-tailed Student’s t-test. (**E**) Cell viability assay of 393P-vec and miR-181c/d OE cells. Statistical significance was determined by two-way ANOVA. (**F**) Transwell migration assay comparing 393P-vec and miR-181c/d OE cells. Statistical significance was determined by two-tailed Student’s t-test. (**G**) qRT-PCR validation of miR-181c and miR-181d levels in 393P-ZEB1 cells transfected with miR-181c/d inhibitors. **P < 0.01 and ***P < 0.001 by two-tailed Student’s t-test. (**H**) Transwell migration assay of 393P-ZEB1 cells transfected with miR-181c/d inhibitors. (**I**) AGO2-RIP assay in 393P-vec and miR-181c/d OE cells. *circDennd1b* co-immunoprecipitated with AGO2 protein was detected by qRT-PCR. (**J**) Luciferase reporter assay in 393P-vec and miR-181c/d OE cells with *circDennd1b* wildtype (WT) and a mutant lacking miR-181-binding site (mut). *P < 0.05 and ***P < 0.001 by two-tailed Student’s t-test.

## Discussion

In this study, we identified *circDennd1b* as a circular RNA suppressed by the EMT transcription factor ZEB1 in lung cancer cells. ZEB1 controls the expression of various circRNAs, including *circDennd1b.* As a tumor suppressor, *circDennd1b* inhibits EMT-associated migration and invasion, partly by sponging the miR-181 family.

ZEB1 regulates various non-coding RNAs to facilitate cancer progression (Ahn et al. 2012; Kim et al. 2022), in addition to the transcriptional repression of epithelial genes such as *CDH1* and miR-200 family (Kalluri and Weinberg 2009). Previously, we found that *lnc-Nr2f1*, a ZEB1-regulated long non-coding RNA, promotes the migration and invasion of lung adenocarcinoma cells (Kim et al. 2022). In this study, we report the ZEB1/*circDennd1b* axis. This shows that ZEB1 drives EMT through the regulation of miRNA, long non-coding RNAs, and circRNAs.

Here, we showed that ZEB1 downregulates *circFbln2*, *circOxr1*, *circHipk3*, *circTlk1*, and *circSlc8a1*, but upregulates *circTut4*. *circHipk3* generally promotes tumor growth, but it can act as a tumor suppressor depending on the contexts (Zhang et al. 2020). *circFbln2* inhibits myoblast proliferation by sponging miR-22-5p (Liu et al. 2025); however, in our data, its downregulation by ZEB1 may contribute to the mesenchymal phenotype in lung cancer. This suggests that ZEB1 broadly changes circRNA expression during cancer progression.

ZEB1 overexpression significantly reduced *circDennd1b* levels without affecting its host gene *Dennd1b*. DENND1B is a guanine nucleotide exchange factor involved in endocytic trafficking and asthma-related immune responses (Yang et al. 2016). While the role of *Dennd1b* in cancer is unclear, *circDennd1b* was recently reported as a tumor suppressor in clear cell renal cell carcinoma by sponging miR-122-5p (Chen et al. 2022). Here, we show that *circDennd1b* inhibits EMT in lung cancer and is directly regulated by ZEB1.

We found that miR-181 family is the main target of *circDennd1b*. These oncogenic miRNAs promote tumor development by inhibiting various tumor suppressors. For example, miR-181a targets SMAD7 to induce EMT in ovarian cancer (Parikh et al. 2014), and targets PTEN to change metabolism in colon cancer (Wei et al. 2014). Also, exosomal miR-181d from cancer-associated fibroblasts increases EMT and tumor growth in breast cancer (Wang et al. 2020). Here, we suggest that the decrease of *circDennd1b* during ZEB1-induced EMT allow miR-181 to promote lung cancer cell invasion. To find downstream targets, we compared our RNA-seq data with TargetScan prediction (Supplementary Figure 3A). Known miR-181 targets like SMAD7 and PTEN did not change in our model, showing that this regulation is context-dependent. Among the predicted targets, the lipid phosphatase MTMR9 was a candidate downstream target (Supplementary Figure 3B-D). MTMR9 knockdown has been reported to increase AKT activation, similar to PTEN loss (Guo et al. 2013). We will investigate the detailed functions of MTMR9 and other candidates in future studies.

It is unclear how ZEB1 selectively inhibits *circDennd1b* circularization without affecting its linear transcript. As a transcription factor, ZEB1 might act indirectly through RNA-binding proteins (RBPs). Using RBPmap (https://rbpmap.technion.ac.il/), we identified potential RBP binding sites in the flanking introns of *circDennd1b*. Among them, three candidate RBPs (ESRP1, YBX2, and EIF4A3) were downregulated by ZEB1 overexpression (Supplementary Figure 4). ESRP1 is a known ZEB1 target that regulates circRNA biogenesis (Liu et al. 2024), and EIF4A3 is involved in ZEB1-dependent *circDOCK5* formation (Meng et al. 2021). Thus, ZEB1 likely controls *circDennd1b* levels by repressing these RBPs. Alternatively, ZEB1 may directly bind to the flanking introns to regulate *circDennd1b*, similar to its effect on *circNIPBL* (Li et al. 2023). Using *in silico* prediction, we found potential ZEB1-binding motifs in these introns of *circDennd1b* (112- and 197-nt upstream of Exon 4 and 11- and 323-nt downstream of Exon 9). We will test these possibilities in future studies. Finally, although we focused on the miR-181 family, *circDennd1b* may also interact with other miRNAs, such as miR-122 (Supplementary Figure 5) (Chen et al. 2022), or specific proteins depending on the cellular context.

Our findings suggest that the ZEB1/*circDennd1b*/miR-181 axis regulates lung cancer progression. Thus, restoring *circDennd1b* expression or inhibiting the miR-181 family could be considered for treating lung adenocarcinoma.

## Supplementary Material

Supplementary Info_circRNA.pdf

## Data Availability

The RNA-seq data discussed in this study have been deposited in NCBI’s Gene Expression Omnibus and are accessible through GEO Series accession number GSE319378.
