# Supplementary material for: ZEB1-mediated suppression of circDennd1b promotes the migration and invasion of lung cancer cells
Source: Anim Cells Syst (Seoul). 2026 Oct 6;30(1):547–57. doi: 10.1080/19768354.2026.2742602 (PMC13647367; doi:10.1080/19768354.2026.2742602)
Supplement: Supplementary file 1 [file TACS_A_2742602_SM7865.pdf]

Supplementary Table 1. The list of primers used in RT-PCR and qRT-PCR

Gene name	Forward (5′→3′)	Reverse (5′→3′)
circDennd1b	CAACATGCTGCGACTGTACG	AGATGTGAGCCTGCAGAACC
circFbln2	AGTAAAGCCCAGCGATCACC	AGCCACACTCCTGCAGATTC
circOxr1	GACCACTTGTATGCCTTCTTCA	TCCTTCTCCCATCTTTCTTGTCT
circHipk3	AGACCAAGACTTGTGAGGCC	GTGATCCGGCCTGTTCTTCA
circTlk1	CTCCAACACTGCAACTCCCA	TCACAGTTTTGGAAGCTTAGGA
circSlc8a1	CCTCCTGTTTCTGCCTCTGT	CACCTGTGGAGAGCTCGAAT
circPeak1	GCATTCGCCAGGTTTCCAAA	GGAGGTGTTACTGCGGAACT
circTut4	GGGAGAAAAGTCAGCTCTGGA	GCAGGGCTCAGGTGTATGAA
Rpl32	ATCAGGCACCAGTCAGACCGAT	GTTGCTCCCATAACCGATGTTGG
Dennd1b	AGCAGTGACCAAAGCAACTCCC	GCTCTGCAGAACTTCCTGGT
Zeb1	ATTCAGCTACTGTGAGCCCTGC	CATTCTGGTCCTCCACAGTGGA
Zeb2	GCAGTGAGCATCGAAGAGTACC	GGCAAAAGCATCTGGAGTTCCAG
Vim	GCGTGCGGCTGCTTCAAGAC	ATGGCGTCGGCCAGCGAGAA
Twist1	TCGACTTCCTGTACCAGGTCCT	CCATCTTGGAGTCCAGCTCG
Twist2	CAGCAAGATCCAGACGCTCAAG	ACACGGAGAAGGCGTAGCTGAG
Cdh1	GGTCATCAGTGTGCTCACCTCT	GCTGTTGTGCTCAAGCCTTCAC
Cdh2	CCTCCAGAGTTTACTGCCATGAC	CCACCACTGATTCTGTATGCCG
Crb3	CGGACCCTTTCACAAATAGCA	CGTTGGACTCATCACCTGGG
Cldn3	CACCTGACTACCGGGCCTAG	GGTTTCTTTGTCCATTCGGCT
Pard6b	TTTCCACCGCCAATCCACTGCT	GCTGATGACGATGTGAGGCTTC
Inadl	GGAAGATTTGCCTCTGTACCGAC	GCTGAAGTTCGGTGTCTCCTCT
Snai1	TGTCTGCACGACCTGTGGAAAG	CTTCACATCCGAGTGGGTTTGG
Snai2	ATCCTCACCTCGGGAGCATA	TGCCGACGATGTCCATACAG
Cd44	CGGAACCACAGCCTCCTTTCAA	TGCCATCCGTTCTGAAACCACG
Wnt9a	AGTGCCAGTACCAGTTCCGCTT	GAGATGGCGTAGAGGAAAGCAG
Mtmr9	AGGTGGCTTTGAACAGGAGGCT	CCACGAGTTTGATTAGGCTCTCC
Ipmk	CTTCACTCTGACAGCTACGAGAC	GAATACTGGCGGCAATCGCATC
Osbpl3	ATCCATGCGGAATCTCTGCTCC	AGACAGCCTGCTCTCATTGGAG
Sowahc	GTATCTGAGCGAGAGCATCGCA	GAGCACCTTTGAAAGTCTCCAGC
Tmem242	CTTTATGCCTGGTGTGGAGTTGG	GGCTGACTCATGGTTCTTGGGA
Jdp2	CACTTCCTGGAGGTGAAACTGG	GCAGCGACTTTGTTCTTTTCCCG
Gpsm1	ACCGAAACGGAGAACTGACAGC	ACTCAGCCTCTGTGTCCGTTTG
Rassf1	GTCCTGAAGGAAAATGACTCGGG	TGCAGGATCTGGCGAAGGTGTT
Pten	TGAGTTCCCTCAGCCATTGCCT	GAGGTTTCCTCTGGTCCTGGTA
Smad7	GTCCAGATGCTGTACCTTCCTC	GCGAGTCTTCTCCTCCCAGTAT
Esrp1	GATGACAGCACAGTGGTGAGAG	CCTGAGCATTCAGGCAAAGTGC
Ybx2	CTATCGAAGGCGGTTTGTGCGA	CCTCCAATGGTGCTGTCTCCTT
Eif4a3	GACCAAAGTGGAGTTCGAGACG	TGATAGCACGCTGCTGAATCGC
Rbm38	CAAGCCTAGGAGCTTACAGACG	GCACAATGGCTGGTGGGTAGAT
Khdrbs1	GGCTATTACAGCCAGAGTCAAGG	TAGCTGGAGGAGCCTTCAGTGA
Pum2	AAGTGCGTCTCTTGGCTGTGGA	GAGACTCTCTCCTTGTGGCACT
Esrp2	TCTCCACCTACTTGAAGGGTCC	TTGTCCACCACATCAGCCTTGC

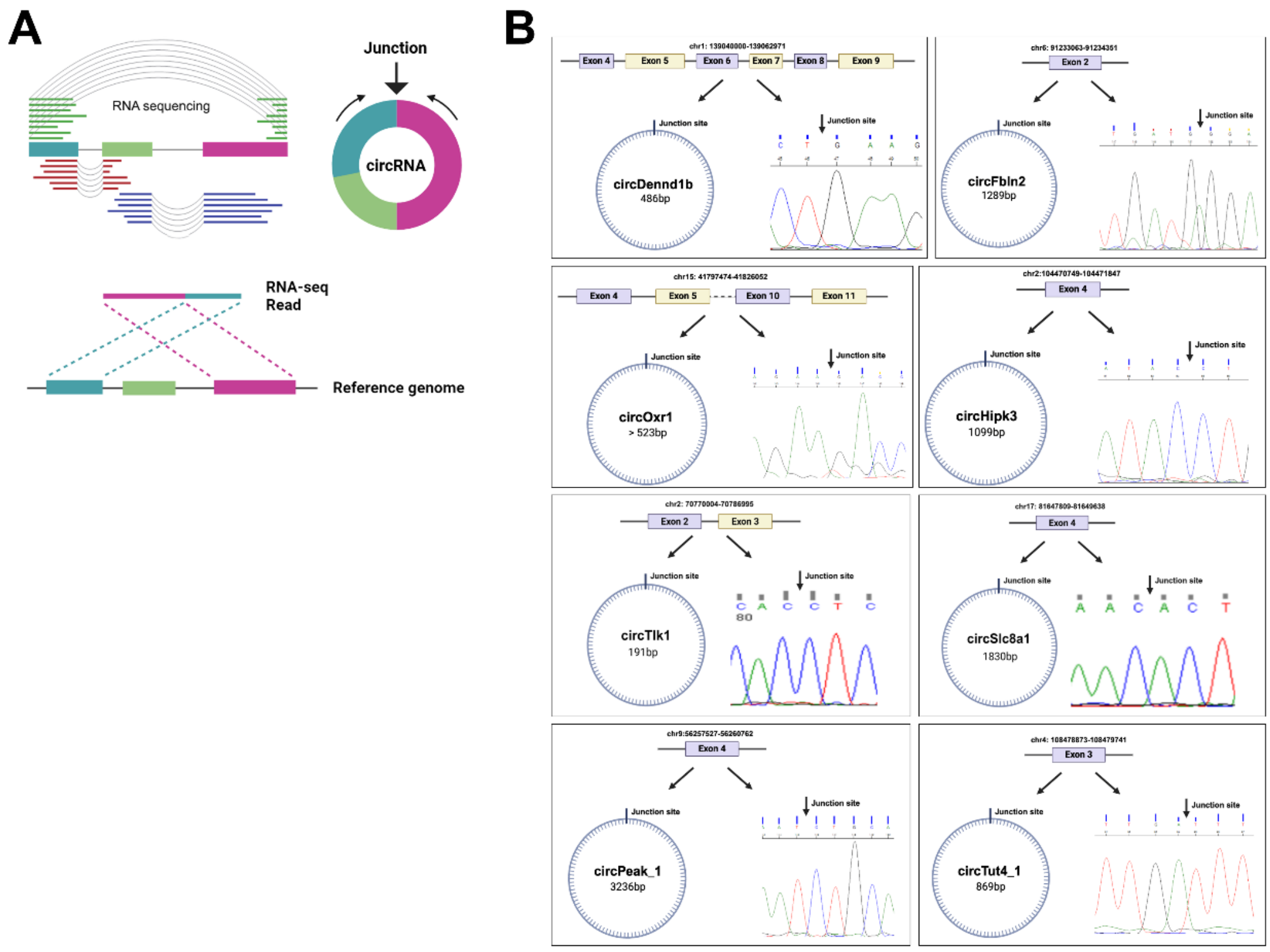

Supplementary Figure 1. Identification and validation of circRNAs

(**A**) Schematic of the bioinformatic pipeline using the CIRI-full algorithm for the reconstruction and quantification of full-length circRNA isoforms from RNA-seq data. (**B**) Sanger sequencing chromatograms of RT-PCR products across the back-splice junction sites, confirming the circular structure of candidate circRNAs

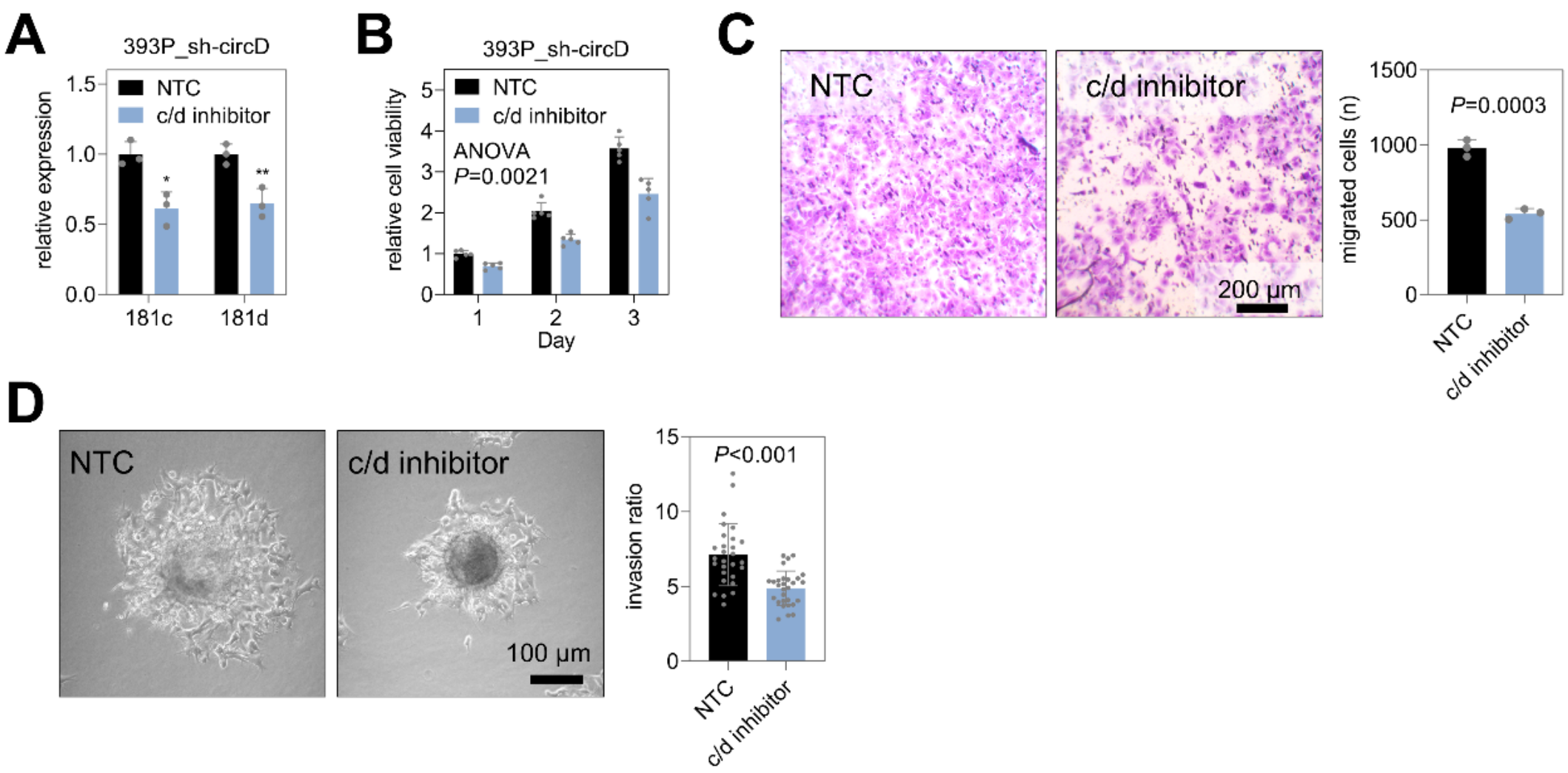

Supplementary Figure 2. *circDennd1b* acts as a sponge for the miR-181 family.

(**A**) qRT-PCR validation of miR-181c and miR-181d levels in 393P-*circDennd1b* knockdown cells (393P_sh-circD) transfected with miR-181c/d inhibitors. $*P < 0.05$, $**P < 0.01$ by two-tailed Student's t-test. (**B**) Cell viability assay of 393P_sh-circD cells transfected with nontargeting control (NTC) or miR-181c/d inhibitors. Statistical significance was determined by two-way ANOVA. (**C**) Transwell migration assay of 393P_sh-circD cells transfected with miR-181c/d inhibitors. (**D**) Spheroid invasion assay of 393P_sh-circD cells transfected with miR-181c/d inhibitors. P-values were determined by two-tailed Student's t-test.

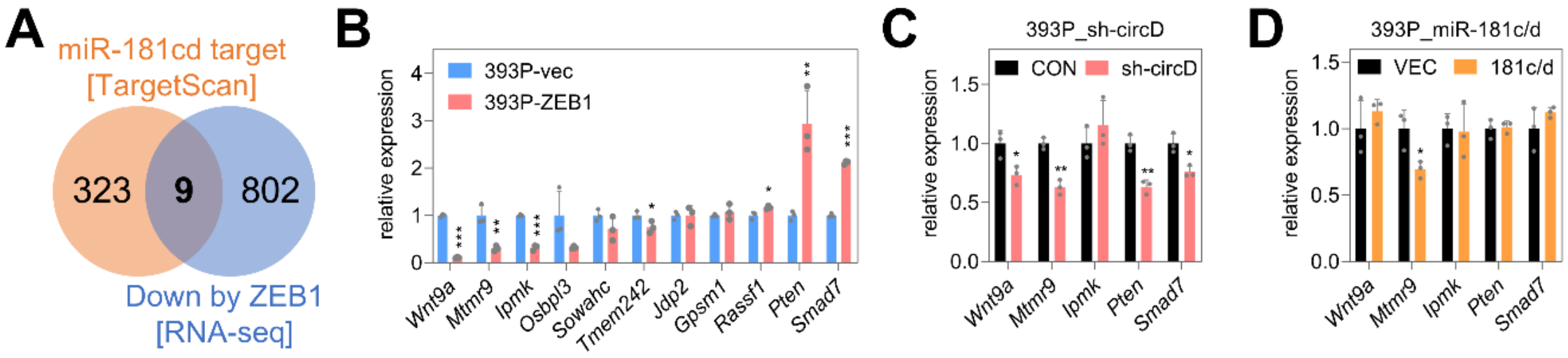

Supplementary Figure 3. Downstream targets of the ZEB1/*circDennd1b/*miR-181 axis

(**A**) Venn diagram overlapping putative miR-181c/d targets (from TargetScan) with ZEB1-downregulated genes (from RNA sequencing). (**B**) qRT-PCR analysis of nine candidate targets, including the previously reported miR-181 targets PTEN and SMAD7. (**C, D**) qRT-PCR validation of candidate genes in 393P_sh-circD and 393P_miR-181c/d overexpressing cells. *P < 0.05, **P < 0.01, ***P < 0.001 by two-tailed Student's t-test.

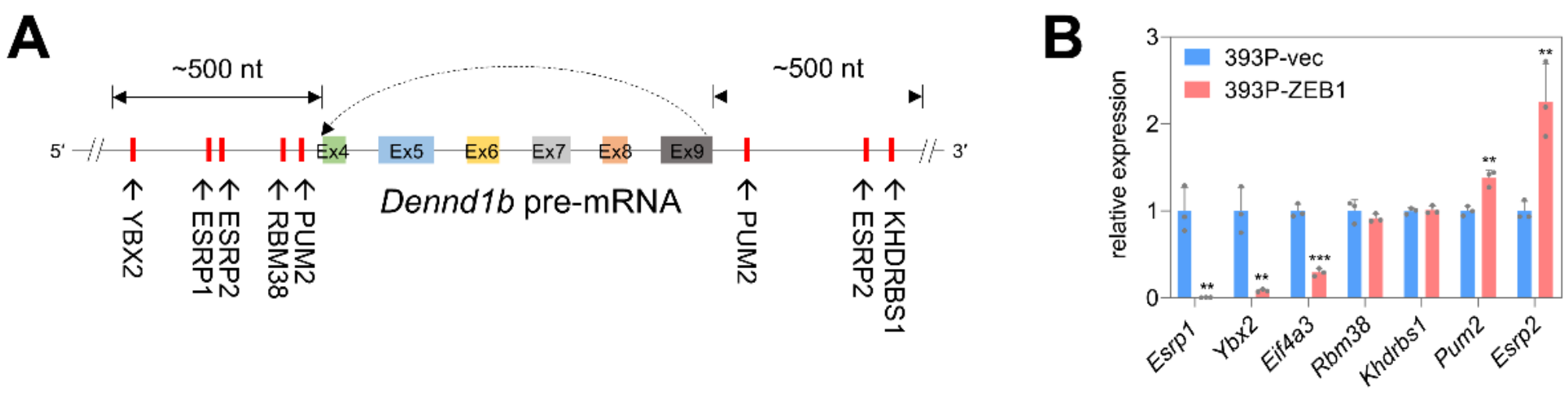

Supplementary Figure 4. Identification of RNA-binding proteins (RBPs) regulated by ZEB1

(**A**) Diagram showing putative RBP-binding sites near the flanking introns of *circDennd1b*. (**B**) qRT-PCR of RBPs in 393P-ZEB1 cells. **P < 0.01, ***P < 0.001 by two-tailed Student's t-test.

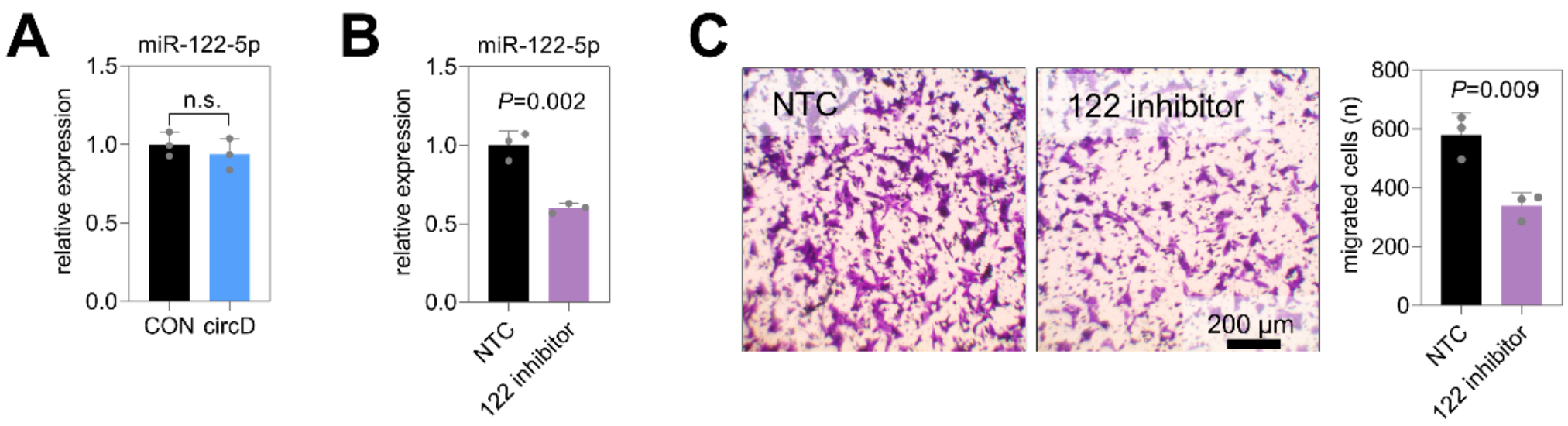

Supplementary Figure 5. The effect of miR-122 on cancer cell migration

(**A**) qRT-PCR of miR-122-5p in 393P-ZEB1_*circDennd1b* overexpressing cells. (B) qRT-PCR of miR-122-5p in 393P-ZEB1 cells transfected with miR-122 inhibitor. (C) Transwell migration assay of 393P-ZEB1 cells transfected with miR-122 inhibitor. P-values were determined by two-tailed Student's t-test.
